# A Splendid Special Hospital

**Published:** 1888-08-11

**Authors:** 


### IN AND OUT AMONG THE HOSPTALS.

By a Secretary of Ten Years' Standing.

Copies of Annual Reports and Rules, Accounts of Meetings, Changes in the Staff, &c., should be addressed The Editor, The Lodge, Porchester Square, W.

### A SPLENDID SPECIAL HOSPITAL.

It has not infrequently been our mission, in the discharge of a plain duty to the public, to direct attention to some of the many defects and abuses attaching to certain special hospitals. It is therefore a real satisfaction, out of fullness of knowledge, based upon the closest examination and inquiry, to be able to say confidently that there is one ideally perfect special hospital in London. Perfect that is as to its genuine character, as to the necessity for its separate existence, as to the excellence of its management, and the singular ability and skill brought to bear upon the administration of its affairs. We commend to our colleagues of the daily Press, to those engaged in the government of hospitals, and especially to the wealthy who support voluntary charities, the report upon the rebuilding of the National Hospital for the Paralysed and Epileptic, Queen Square, with a statement of receipts and expenditure (The Chiswick Press, Chancery Lane), the special hospital to which we refer. This brief brochure is worthy of careful study, for it contains in twelve short pages the records of one of the best pieces of work ever done by a hospital committee. What is even more to the purpose, the whole enterprise has been carried through from first to last with a rapidity, a careful husbanding of resources, and an expenditure upon appeals, advertisements, and postage so small as to practically represent the irreducible minimum in such matters. Internally, too, this hospital is worthy of a visit, as it exhibits in a marked degree what may be done to beautify and enliven the wards and offices of a hospital without appreciable extra outlay. Possibly the spirit which animated one of England's noblest princes, the late Duke of Albany—to whom the new buildings are dedicated, by permission of the Queen—may have entered into those who have had to do with the National Hospital in these latter days. Be this so or not, the National Hospital for the Paralysed and Epileptic, as at present conducted, is one we may all be proud of, from the Queen downwards, and many might do worse than spend a spare hour in calling upon the able and devoted secretary and director, Mr. B. Burford Rawlings, to whom the committee and governors owe so much of their success. Paying patients are admitted to one portion of the hospital, a fact which persons suffering from nervous diseases, and their friends, will do well to make a note of, for there are few more comfortable abodes for such cases than the National Hospital in Queen Square.

### THE GENERAL HOSPITAL, BIRMINGHAM.

The condition of the General Hospital, Birmingham, one of the oldest and best conducted of provincial institutions, is anything but encouraging from a financial point of view, the income for the last three years having been steadily on the decline. An old debt of £3,971 was increased last year to £6,847, as shown by the report, but the most unfavourable sign is the serious falling off of £210 in annual subscriptions. It is true that the Birmingham musical festival will be held next year, and that the proceeds will be devoted to this hospital, but it is too much to hope the amount received—usually about £4,000—will not make up the deficiency. The total receipts for the last twelve months were £12,217, and the expenditure £15,020. Deducting 1*s.* for each out patient, the expenditure on structural alterations, and the money expended for Samaritan or convalescent purposes it is found that the cost per bed occupied is £49 17*s.* 2*d.*, there having been a daily average of 251 patients in the wards. The in-patients numbered 3,859, and the out-patients 47,941. Mr. Henry Fox, R.N., resigned his appointment as house governor and secretary during 1887, and Dr. M. Coghill has been appointed house governor. The Jaffray Branch Hospital, the first chronic hospital established in this country, and one which Birmingham owes to the munificence of one of her most respected public men, Mr. John Jaffray, J.P., which is situated in the suburbs of Birmingham, but under the same management as the General Hospital, appears to be in a more flourishing condition. 182 patients were treated during the year, and the Duke of Norfolk opened the fourth wing a short time ago. The Jaffray Hospital is too little known as yet to be appreciated as it deserves, but other large towns are sure to establish chronic hospitals on similar lines before many years are over.

### THE WEST LONDON HOSPITAL.

After considerable alteration, the West London Hospital is again fulfilling its mission in the thickly-populated neighbourhood in which it is situated. Although there are many special hospitals in the S.W. district, there is no other general hospital westward of Hyde Park Corner, and as there is an enormous traffic passing its doors, a large number of surgical beds are kept constantly occupied. It is gratifying to find that the hospital has gained the confidence of those interested in it, as is shown by a very considerable increase in the receipts. The total income for the year was £5,892 9*s.* 8*d.*, and the expenditure £5,386 13*s.* 3*d.*; the cost per bed occupied, after deducting the extraordinary expenditure and 1*s.* for each out-patient, is £56 5*s.* 11*d.*, a decidedly low rate for a metropolitan general hospital. The patients numbered 1,223 in the wards and 16,522 in the out-patient department. The average period of residence of each in-patient was 23 days, and the average number of beds occupied 81. An important change in the work of the out-patient department has been made by the opening of four more special departments, viz., aural, cutaneous, orthopædic, and diseases of the throat and nose, which have been put under the charge of Mr. Wainwright, Dr. Drewitt, Mr. Keetley, and Dr. Ball respectively. An electrical department for both in and out patients has also been added, under the care of Dr. Herringham. The committee record their indebtedness to the Honorary Solicitor, Mr. Bingham Watson, who has conducted all the law business of the hospital, which last year was very great, entirely free of cost. This is the hospital to which *Truth* declares, on the authority of its chairman, Mr. Stephen Paget was elected an assistant-surgeon because H.R.H. the Prince of Wales, as patron of the hospital, said he would be personally obliged to the directors if they would so elect him. We do not believe a word of it.

### ROYAL LONDON OPHTHALMIC HOSPITAL.

This is the largest eye hospital in the United Kingdom, and probably treats a greater number of patients than any other similar hospital. So great is its reputation that patients are sent to Moorfields from very great distances, many coming even from abroad. It has long been felt that the bed accommodation is inadequate to the requirements of those who seek admission. To the poor their eyesight is all important, and if taken in time most derangements of the eye are amenable to treatment in the hands of a skilled surgeon. The building is old, and many of the defects are apparent and need attention. An effort is therefore being made to put the hospital in a thoroughly sanitary condition, and at the same time to provide further bed accommodation. For this purpose the managers make an appeal to the public, which will not, we hope, be made in vain. The income for the year was £4,400, and the expenditure £5,918. The cost per bed occupied was £50 8s. 2d. The total number of patients was 26,732, of which 2,185 were in-patients. The daily average number was 93.

